# Multimodal Gait Acquisition and Musculoskeletal Simulation for Subject-Specific Evaluation of Ankle Exosuit Cable Anchor Positioning in Cerebral Palsy

**DOI:** 10.3390/biomimetics11100731

**Published:** 2026-10-13

**Authors:** Javier Bermejo-García, Alberto Martín-Fraile, Joaquín Ojeda, Rafael Agujetas, Francisco Javier Alonso Sánchez

**Affiliations:** 1 Department of Biomechanical Engineering, University of Twente, 7522 NB Enschede, The Netherlands; javier.bermejo@utwente.nl; 2 Department of Mechanical Engineering, Energy and Materials, University of Extremadura, 06006 Badajoz, Spain; albertomf@unex.es (A.M.-F.); fjas@unex.es (F.J.A.S.); 3 Department of Mechanical Engineering and Manufacturing, Higher Technical School of Engineering, University of Seville, 41092 Seville, Spain; joaquinojeda@us.es

**Keywords:** multimodal gait sensing, motion capture systems, ankle exosuit, musculoskeletal simulation, metabolic cost modeling, wearable assistive devices

## Abstract

Wearable ankle exosuits require precise evaluation frameworks to optimize assistive force transmission and subject-specific configuration. This study presents a multimodal sensor-based experimental and computational framework to assess the influence of ankle exosuit cable anchor positioning on neuromuscular and energetic responses during gait in a child with spastic hemiplegic cerebral palsy. Three-dimensional kinematics were acquired using an optical motion capture system (12 infrared cameras), while ground reaction forces were recorded through instrumented force plates. Metabolic cost was experimentally measured using portable respiratory gas analysis. These sensor-derived data were integrated into a participant-scaled musculoskeletal model in OpenSim to simulate nine cable anchor configurations and quantify changes in muscle activation, force production, mechanical impulse, and metabolic power. Cable geometry altered the predicted distribution of plantar-flexor loading across configurations. Experimental measurements were not available for muscle-level outputs or for the assisted configurations; therefore, these responses were interpreted as model-derived, exploratory comparisons rather than experimentally validated physiological effects. Configurations with larger offsets from the heel-reference position tended to yield greater reductions in gastrocnemius demand, whereas configurations nearer the heel reference more effectively reduced soleus loading but increased secondary stabilizing muscle recruitment. Predicted metabolic changes across assisted configurations ranged from a 0.86% reduction to a 6.28% increase relative to the experimental baseline, and from a 2.66% reduction to a 4.33% increase relative to the simulated non-actuated condition. These differences should be interpreted as indicators of model sensitivity rather than demonstrated energetic improvements. This study demonstrates how multimodal gait sensing combined with musculoskeletal simulation provides a quantitative tool for preliminary screening of cable-driven ankle exosuit design in pathological gait.

## 1. Introduction

Cerebral palsy (CP) is a non-progressive neuromotor disorder that affects motor control, gait, and energy efficiency, leading to altered muscle activation patterns and increased metabolic demand during walking [1,2]. The ankle plays a central role in gait progression, push-off, and balance control, functions that are frequently compromised in children with CP because of impaired neuromuscular control, muscle weakness, joint stiffness, and restricted range of motion [3,4]. These biomechanical impairments are associated with less efficient gait and greater energy expenditure [1,3,5]. Consequently, assistive strategies targeting ankle function may contribute to reducing muscular demand and improving walking functionality in children with CP [1,4].

In recent years, textile-based and cable-driven ankle assistive devices, commonly referred to as exosuits, have emerged as lightweight alternatives to rigid exoskeletons [6,7]. Their compliant structure may offer improved compatibility with natural movement, reduced distal mass, and greater comfort during prolonged use. Previous studies have reported changes in metabolic cost, muscle activation, and spatiotemporal gait parameters in healthy participants and in individuals with neurological impairments, particularly after stroke [6,7,8]. Exosuit-assisted rehabilitation programs have also shown potential to increase ankle propulsion and community walking activity [9,10]. Nevertheless, the clinical evidence remains limited, particularly in pediatric neurological populations, and further controlled studies are required to determine their long-term effectiveness and the extent to which their benefits can be generalized across patients [11,12].

The mechanical performance of a cable-driven exosuit depends not only on the magnitude and timing of the applied assistance, but also on the geometry of the cable path and the location of its attachment points. Changes in anchor position modify the cable moment arm, force direction, and load transmission to the user. Consequently, two configurations delivering the same nominal ankle assistance torque may produce different muscle-loading patterns and may redistribute demand among primary plantar flexors and secondary stabilizing muscles. Experimental evaluation of numerous cable configurations is, however, costly and difficult, particularly in children with motor impairments. Computational simulation therefore provides a useful preliminary tool for comparing design alternatives before the manufacture and testing of physical prototypes.

Musculoskeletal simulation has been widely used in the development and assessment of orthoses, exoskeletons, and exosuits [13,14]. By integrating experimentally measured kinematics and external forces, these models can estimate joint moments, muscle activations, muscle forces, and metabolic variables that are difficult to measure directly in vivo [15]. Such simulations do not replace experimental validation, but they can support controlled comparisons in which selected design parameters are varied while the remaining biomechanical conditions are kept constant. This is particularly useful for investigating the isolated mechanical influence of cable geometry.

Metabolic cost is commonly used in human movement biomechanics and rehabilitation engineering as an indicator of locomotor efficiency during walking, running, cycling, and other physical activities [16,17,18,19,20,21]. It has also become an important criterion for evaluating the design and control of assistive technologies [22,23] and for characterizing pathological gait [18,23,24]. Within musculoskeletal simulations, metabolic models provide an integrative estimate of muscular energy demand. However, their predictions depend on model assumptions, muscle parameters, and the available experimental calibration data. Therefore, small differences between simulated configurations should be interpreted primarily as indicators of model sensitivity rather than as direct evidence of clinically meaningful energetic improvement.

Recent research on wearable robotic assistance has increasingly combined experimental gait analysis and computational modeling to investigate human–device interaction and the influence of assistive forces on muscle and joint mechanics [25,26]. Nevertheless, limited information is available on how the distal cable attachment location of an ankle exosuit affects the predicted distribution of muscular demand in pediatric CP gait. In addition, methodological descriptions often focus on a particular device or participant without clearly identifying the experimental and computational requirements needed for the approach to be reproduced by other research groups.

Accordingly, the present study introduces a proof-of-concept experimental– computational workflow for comparing ankle exosuit cable-anchor configurations in a child with spastic hemiplegic CP. Three-dimensional motion capture, ground reaction forces, and baseline respiratory measurements were integrated into an anthropometrically scaled musculoskeletal model. Nine distal cable-anchor configurations were then evaluated under identical prescribed kinematics and a fixed assistance level. The purpose of this controlled comparison was to isolate the influence of cable geometry on predicted plantar-flexor activation, muscle force, mechanical impulse, and metabolic power.

The study did not seek to identify a universally optimal anchor position or to establish clinical efficacy. Rather, it examined whether the proposed framework can distinguish configuration-dependent mechanical responses within an individual case and provide information for the preliminary screening of cable-routing alternatives. The workflow is intended to be transferable to other research groups provided that synchronized three-dimensional kinematics and external force data, a suitable musculoskeletal model, a defined assistive torque profile, and explicit cable-path geometry are available. Experimental assisted trials, electromyographic measurements, repeated gait cycles, and uncertainty analyses would additionally be required before the predicted responses could be considered physiologically validated or generalized to a wider population.

We hypothesize that, under identical kinematic and assistance conditions, changes in distal cable-anchor position will modify the predicted distribution of muscular demand rather than producing a uniform reduction across all plantar-flexor muscles. The resulting comparisons are expected to clarify the trade-offs between unloading the principal plantar flexors and increasing the contribution of secondary muscles, while demonstrating the potential and limitations of the proposed workflow as a preliminary design-assessment tool.

## 2. Materials and Methods

The methodological workflow comprised two main stages. First, experimental gait data were acquired to obtain the participant-specific kinematic and kinetic inputs required for the simulations. Second, the recorded data were processed and integrated into an OpenSim musculoskeletal model to estimate muscle activation, muscle force, and metabolic power under the different simulated cable-anchor configurations.

### 2.1. Data Acquisition Workflow

Gait data were collected from a pediatric participant with cerebral palsy in order to evaluate, within a controlled simulation framework, the mechanical influence of cable-driven ankle assistance. The participant was a 12-year-old child with a body mass of 44 kg and a height of 1.47 m, diagnosed with right-sided spastic hemiplegia. The participant exhibited a functional but impaired gait pattern associated with reduced neuromuscular control of the muscles acting on the right ankle. During habitual walking, the participant used a clinically prescribed passive ankle–foot orthosis (AFO) on the affected limb to limit excessive plantar flexion and internal rotation.

Written informed consent was obtained from the participant’s legal guardian before data collection. The experimental protocol was conducted in accordance with institutional ethical requirements and was approved by the Ethics Committee for Research of the Servicio Extremeño de Salud and by the Bioethics Committee of the Universidad de Extremadura (approval number 10/2023).

The participant walked at 4km/h along an instrumented walkway while wearing the passive AFO used during habitual gait. Three-dimensional marker trajectories were recorded using 12 infrared optical cameras (OptiTrack) operating at a sampling frequency of 100 Hz. A total of 28 reflective markers were attached to the participant according to the anatomical marker protocol proposed by [27]. Ground reaction forces were recorded using two force plates (Bertec *^TM^* FP-4060, Bertec Corporation, Columbus, OH, USA) at a sampling frequency of 1000 Hz.

A static calibration trial was first acquired for anthropometric scaling of the musculoskeletal model. Subsequently, several walking trials were recorded for the dynamic analysis. Only trials containing complete foot contact on both force plates without targeting effects were considered valid for further analysis. Among these valid trials, the gait cycle exhibiting the highest continuity of marker trajectories and the lowest number of interpolated frames was selected for the musculoskeletal simulations.

The gait trial therefore represented the participant’s walking pattern while using the clinically prescribed passive AFO and should not be interpreted as unassisted pathological gait without orthotic support.

The recorded marker trajectories and ground reaction forces were processed using a fourth-order low-pass Butterworth filter with a cutoff frequency of 6 Hz. The processed kinematic and kinetic data were subsequently used as inputs to the OpenSim simulation workflow.

**Figure 1:**
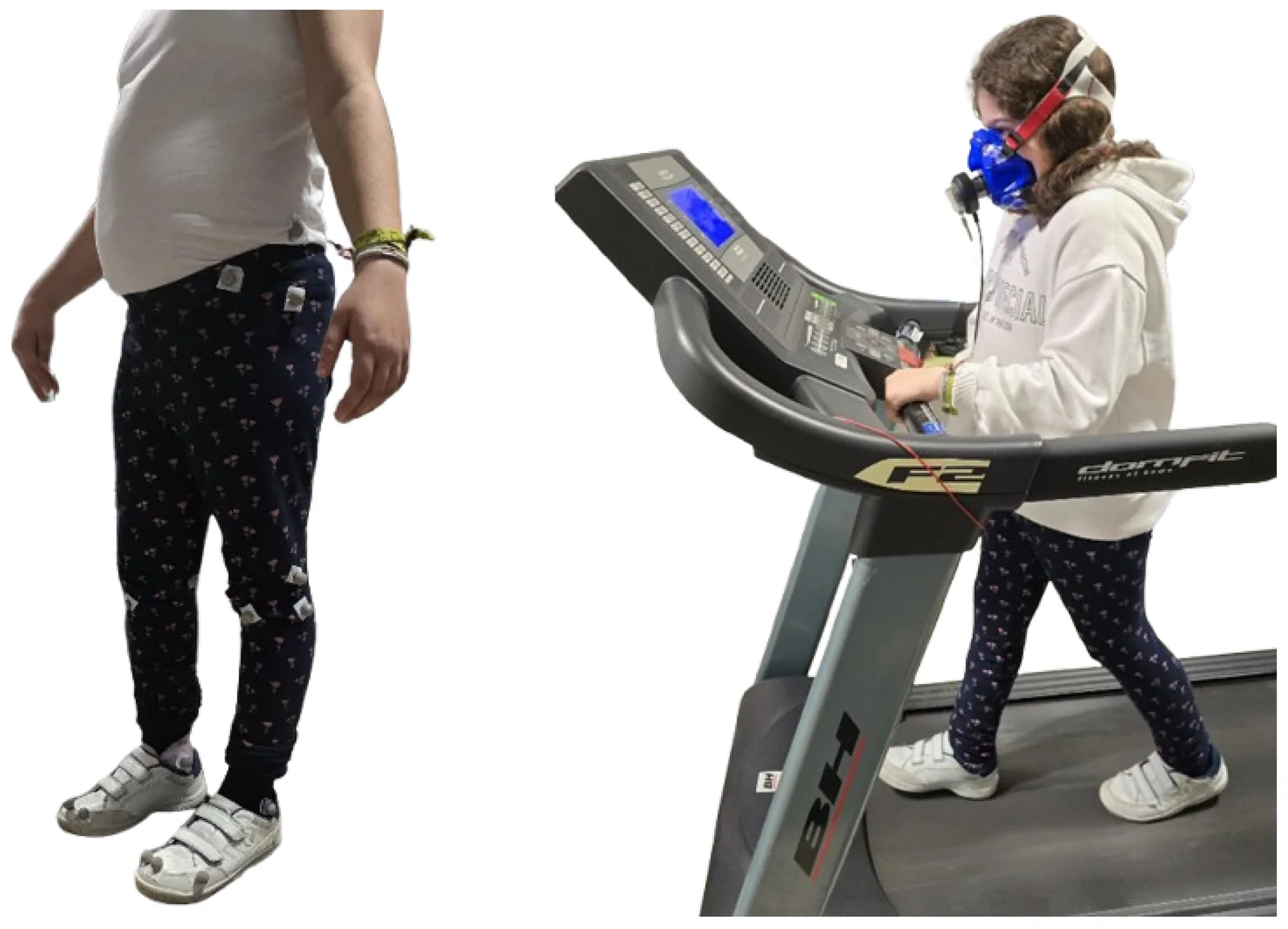
Experimental data acquisition. (**Left**): participant wearing the anatomical marker set used for three-dimensional motion capture. (**Right**): treadmill walking during respiratory gas analysis.

Experimental metabolic power was also estimated by indirect calorimetry using a portable respiratory gas analyzer (Q-NRG^®^, COSMED Srl, Albano Laziale (Rome), Italy). Respiratory measurements were obtained during treadmill walking at 4km/h, matching the overground walking speed used during motion capture (also 4km/h), as illustrated in Figure 1. The participant maintained this constant treadmill speed for 7 min. The respiratory gas analyzer provided measurements every 30 s, and steady state was considered to have been reached when the final consecutive measurements showed no appreciable variation, indicating a stable respiratory plateau. Although walking speed was identical in both protocols, treadmill and overground walking represent different experimental conditions; therefore, the metabolic measurement was used as a baseline physiological plausibility check rather than as a direct trial-by-trial validation of the overground simulation.

These measurements were obtained only for the baseline condition and were used to assess the physiological plausibility of the metabolic estimates generated by the musculoskeletal model. No experimental metabolic measurements were available for the simulated exosuit-assisted conditions; therefore, metabolic changes predicted for the assisted configurations were treated as exploratory, model-based comparisons rather than experimentally validated reductions in metabolic cost.

The present study was designed as a single-subject proof-of-concept investigation. Its purpose was not to characterize population-level variability in cerebral palsy or to establish clinical effectiveness, but rather to examine whether the proposed experimental–computational workflow could detect configuration-dependent differences under controlled modeling conditions. The results should therefore be interpreted as subject-specific model predictions rather than as experimentally validated or generalizable effects of ankle exosuit assistance.

### 2.2. Musculoskeletal Model

Computational musculoskeletal models provide a useful complementary tool for the preliminary evaluation of assistive walking devices and for the investigation of biomechanical variables that are difficult to measure directly in vivo, such as individual muscle forces and muscle metabolic power.

The model used for the simulations was the *Gait2392 Simbody* model provided by OpenSim [28,29]. This model comprises 23 degrees of freedom and 92 muscle–tendon actuators. Although the model was originally developed to represent the musculoskeletal geometry of healthy adults, its use as a generic template is common in preliminary simulation studies and controlled design comparisons.

The musculoskeletal geometry was adapted to the participant using the standard marker-based scaling procedure implemented in OpenSim. A static calibration trial was acquired with the 28-marker set positioned over palpable anatomical landmarks according to Leardini et al. [27]. The generic Gait2392 segment dimensions and inertial properties were scaled so that the virtual-marker locations reproduced the experimental marker geometry as closely as possible. The OpenSim Scale Tool reported an overall root-mean-square (RMS) marker-position error of 2.56cm (total squared marker error $=0.017\;m^{2}$). This value is reported as a quantitative measure of the marker-based geometric fit and should not be interpreted as a direct measure of joint-center or muscle-path accuracy. No additional functional joint-center calibration, medical-imaging-based skeletal reconstruction, or subject-specific redefinition of joint axes, muscle attachment sites, or wrapping surfaces was performed. Consequently, the scaled model preserved the underlying skeletal topology and muscle-path definitions of Gait2392 and did not capture possible participant-specific skeletal deformities or altered muscle geometry associated with cerebral palsy. Likewise, no additional modifications were introduced to represent spasticity, muscle contractures, selective muscle weakness, or modified muscle–tendon mechanical properties.

The muscle–tendon units followed the Hill-type muscle model proposed by [30]. The default muscle parameters in the generic model are defined using population-averaged values and may therefore differ from the actual physiological properties of the participant. In the present study, the only physiological adaptation applied to the muscle model consisted of scaling the maximum isometric force $F_{iso}^{M}$ of each actuator according to the participant body mass $\left(m_{subj}\right)$ and the reference mass of the generic model $\left(m_{gait2392}\right)$, following the relationship proposed by [31]:

(1) $$F_{m,subj}^{M}=F_{m,gait2392}^{M}\cdot {\left(\frac{m_{subj}}{m_{gait2392}}\right)}^{\frac{2}{3}}$$

This procedure partially adapts the muscle strength capacity of the model to the anthropometric characteristics of the participant while maintaining the original muscle architecture and physiological assumptions of the Gait2392 model. No additional calibration of individual muscle weakness or pathological muscle properties was performed.

OpenSim probes are model components designed to compute additional biomechanical quantities during simulation. In the present study, a metabolic probe was incorporated to estimate energy expenditure. The metabolic energy rate $\left(\dot{E}\right)$ was computed using the muscle energy model proposed by [32], which defines muscle metabolic power as the sum of heat generation and mechanical work rates:

(2) $$\dot{E}=\dot{B}+\sum_{i=1}^{N}\left(\dot{A_{i}}+\dot{M_{i}}+\dot{S_{i}}+\dot{W_{i}}\right)$$

where $\dot{B}$ is the basal heat rate, $\dot{A}$ represents the activation heat rate, $\dot{M}$ is the maintenance heat rate, $\dot{S}$ corresponds to the shortening heat rate, $\dot{W}$ is the mechanical work rate, and *N* is the total number of muscles included in the model. All quantities are expressed in watts.

The metabolic contributions of all muscles were aggregated and integrated over time. The resulting value was divided by the gait cycle duration *T* and normalized by the participant body mass *M* to obtain the average metabolic power (W/kg), according to:

(3) $$E=\frac{1}{MT}\int_{0}^{T}\sum_{i=1}^{N}{\dot{E}}_{i}\left(t\right)\;dt$$

Experimental metabolic cost (MC) was estimated from respiratory measurements obtained using a portable ergospirometer, which directly measures oxygen uptake $\left(VO_{2}\right)$ and carbon dioxide production $\left(VCO_{2}\right)$. Energy expenditure was computed using the indirect calorimetry model originally proposed by [33]. Specifically, metabolic power was calculated according to Equation (4), which expresses metabolic rate as a linear combination of $VO_{2}$ and $VCO_{2}$. The measured values, expressed in mL/L/kg, were converted into metabolic power (W) according to:

(4) $$MC=(3.491\cdot VO_{2}+1.106\cdot VCO_{2})\cdot 0.048$$

where the coefficients represent the energetic equivalents of oxygen and carbon dioxide, respectively. This approach allowed estimation of the average metabolic power during the analyzed walking task.

The experimentally measured metabolic cost was available only for the baseline non-assisted condition and was therefore used as a baseline comparison for the simulated metabolic estimates obtained with the different energy models. Consequently, the metabolic predictions generated for the assisted conditions should be interpreted as relative model-based comparisons between cable configurations rather than as experimentally validated absolute metabolic values.

Similarly, the muscle activation and muscle force outputs obtained from the simulations were not validated against electromyographic recordings or other physiological measurements. Therefore, the predicted muscular responses should be interpreted as model-derived estimates that enable controlled comparisons between simulated configurations under identical kinematic conditions rather than as direct measurements of neuromuscular behavior.

### 2.3. Experimental Data Processing and Simulation

After preprocessing the experimental data, the simulation workflow commenced by scaling the geometry of the generic musculoskeletal model using participant-specific anthropometric scaling to align the virtual markers with the experimentally recorded marker positions. During this procedure, the static calibration trial was used to minimize the spatial discrepancies between experimental and virtual markers. The resulting scaled model was subsequently employed for all simulations.

Joint kinematics were then estimated through inverse kinematics (IK). This procedure minimizes the weighted sum of squared marker errors to identify the model configuration that best reproduces the experimental marker trajectories. To improve the robustness of the solution, higher tracking weights were assigned to anatomical markers than to technical tracking markers.

The resulting joint kinematics were prescribed to the musculoskeletal model in order to reproduce the participant’s experimentally recorded baseline gait pattern. Prescribing joint kinematics allowed muscle force and metabolic estimates to be compared under controlled biomechanical conditions while isolating the mechanical influence of the simulated exosuit assistance. By maintaining identical kinematics across all simulated conditions, the predicted differences between configurations primarily reflect the isolated mechanical effect of cable geometry and force transmission rather than modifications in gait strategy or motor adaptation.

This approach should not be interpreted as a representation of the neuromotor adaptations that may occur during real exosuit-assisted walking. In practice, individuals may alter joint kinematics, muscle recruitment patterns, and gait strategy in response to external assistance. Such adaptive mechanisms were intentionally excluded from the present framework in order to enable controlled comparisons between cable configurations.

Inverse dynamics analysis was subsequently performed using the computed joint kinematics and experimentally measured ground reaction forces to estimate the net forces and moments acting at each joint during gait. The ankle joint moment obtained from inverse dynamics was then used to calculate the cable force required to generate the desired assistive torque. The cable force was estimated as the ratio between the target ankle moment and the cable moment arm, defined as the perpendicular distance between the ankle joint center and the cable line of action [34].

To reduce the residual forces and moments acting on the pelvis due to inconsistencies between experimental measurements and the musculoskeletal model [29,35], the *Residual Reduction Algorithm* (RRA) tool was employed. For each trial, the RRA was executed twice: first to obtain an adjusted dynamic model and subsequently to generate corrected kinematics with improved dynamic consistency.

Muscle-driven simulations were then performed using the *Computed Muscle Control* (CMC) tool [36]. The objective of CMC is to estimate one possible set of muscle excitations capable of reproducing the prescribed motion while satisfying the dynamic constraints of the model. The estimated muscle excitations were not constrained or validated using electromyographic measurements and should therefore be interpreted as model-derived predictions rather than direct representations of physiological neural activation patterns.

Finally, the resulting simulation framework was used to compare multiple exosuit configurations by varying the distal cable anchor position while maintaining identical kinematic and assistance conditions across simulations. This strategy enables relative comparisons between configurations under controlled conditions and can be reproduced in other populations provided that synchronized kinematic data, ground reaction forces, an appropriate musculoskeletal model, and an explicit description of the cable path are available.

#### Cable Force

The simulated exosuit assistance was defined from the net ankle joint moment obtained by inverse dynamics during the participant’s recorded gait while wearing the clinically prescribed passive ankle–foot orthosis (AFO). Because the mechanical contribution of the AFO was not modeled separately, this inverse-dynamics moment should not be interpreted as the isolated biological plantar-flexor demand. Instead, it was used as a common mechanical reference for the controlled comparison of cable-anchor configurations.

The marker trajectories and ground reaction forces used for inverse dynamics were processed with the fourth-order low-pass Butterworth filter described above. According to the OpenSim sign convention used in the simulations, the plantar-flexion component corresponded to the negative portion of the net ankle moment. A non-negative plantar-flexion reference moment was therefore defined as

(5) $$\tau_{PF}\left(t\right)=\max\left[0,-\tau_{ankle}\left(t\right)\right],$$

where $\tau_{ankle}\left(t\right)$ is the net ankle moment obtained from inverse dynamics during AFO-supported gait. The target assistive torque was then prescribed as:

(6) $$\tau_{assist}\left(t\right)=\alpha \;\tau_{PF}\left(t\right),$$

with α=0.30. Thus, assistance was active only during the plantar-flexion portion of the gait cycle and corresponded to 30% of the instantaneous plantar-flexion component of the net ankle moment, rather than to 30% of an isolated biological ankle moment. The selected assistance level was based on our previous work [37] and was maintained for all configurations to isolate the effect of cable geometry.

For each cable-anchor configuration, the instantaneous Path Actuator moment arm about the ankle joint was calculated throughout the gait cycle using the OpenSim computeMomentArm function. The cable force was therefore recomputed from the instantaneous, configuration-dependent moment arm according to

(7) $$F_{cable}\left(t\right)=\alpha \;\frac{\tau_{PF}\left(t\right)}{\left|r(t)\right|},$$

where r(t) is the instantaneous Path Actuator moment arm about the ankle joint. Consequently, the same target ankle-assistance torque profile was used for all configurations, whereas the required tensile-force history differed according to cable geometry.

The dimensional force profile was subsequently normalized by its maximum value to define the excitation profile supplied to the OpenSim PrescribedController:

(8) $$u_{f}\left(t\right)=\frac{F_{cable}\left(t\right)}{\max_{s\in T}F_{cable}\left(s\right)},$$

where $u_{f}\left(t\right)$ is the normalized control signal and $\max_{s\in T}F_{cable}\left(s\right)$ is the maximum dimensional cable force over the gait cycle *T*. The actuator force capacity was set consistently with the dimensional force profile so that normalization of the control signal did not alter the intended tensile-force magnitude.

**Figure 2:**
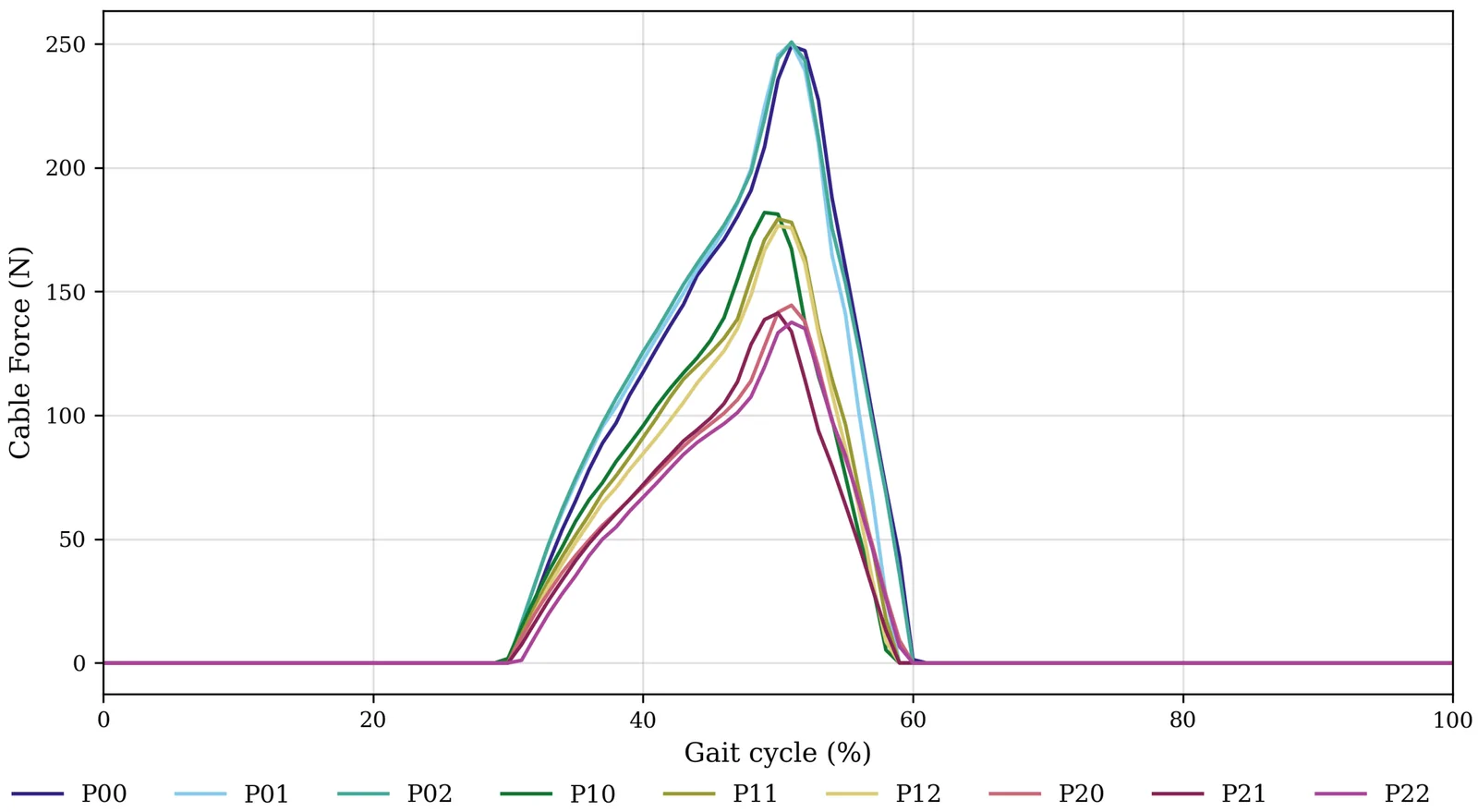
Dimensional cable-force profiles obtained from Equation (7) for the nine evaluated anchor configurations. For each configuration, force was recomputed throughout the gait cycle from the prescribed assistive torque and the instantaneous Path Actuator moment arm.

The cable action was modeled as an idealized tensile force applied between predefined anchor points. This simplified representation enables isolation of the geometric effects associated with cable routing and anchor placement while maintaining identical assistance conditions across all simulations. However, it neglects several relevant aspects of human–device interaction, including textile compliance, soft tissue deformation, dynamic migration of attachment points throughout the gait cycle, friction effects, hysteresis phenomena, and mechanical transmission losses. Consequently, this formulation should be regarded as a conceptual simplification intended to support preliminary design comparisons rather than as a physically realistic representation of the exosuit–human interface (Figure 2).

The actuator was modeled as tension-only, consistent with cable-driven exosuit formulations in which tensile, but not compressive, loads are transmitted [37,38]. Inspection of the complete force histories in the present simulations showed smooth profiles without abrupt force spikes or discontinuities, and no numerical force singularity indicative of a near-zero effective moment arm was observed within the active assistance interval. Across all the configurations, the largest sample-to-sample force variation was approximately 2.37 N. Peak and root-mean-square (RMS) forces are reported in Table 1. Peak force ranged from 137.81 N (P22) to 251.09 N (P02), whereas RMS force ranged from 43.43 to 81.21 N.

**Figure 3:**
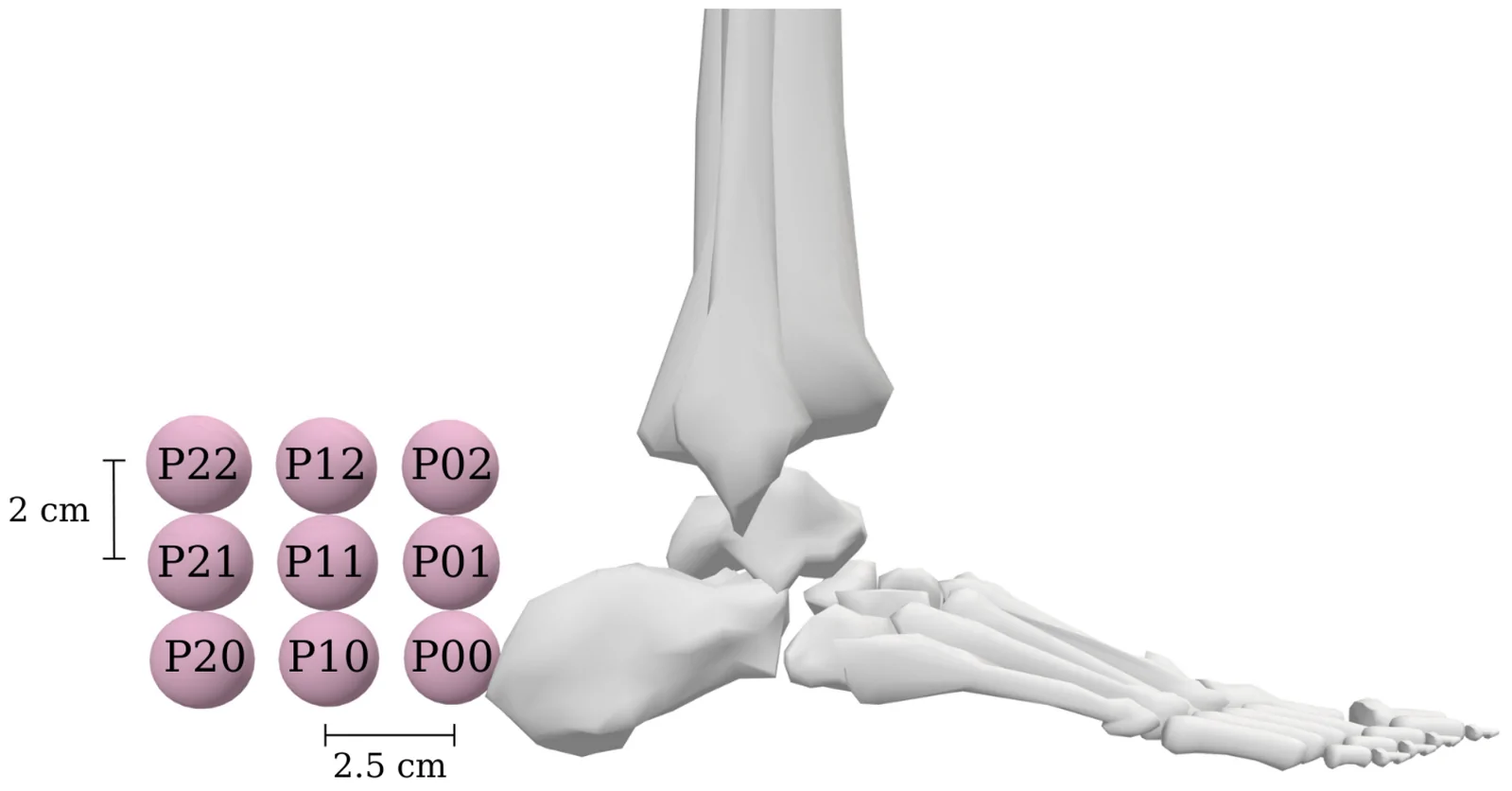
Representation of the nine distal anchor positions evaluated in the musculoskeletal model. P00 is the reference heel-marker position; in the Pij notation, the first index denotes posterior increments of 2.5 cm and the second index denotes superior increments of 2.0 cm relative to P00. Thus, for example, P01 corresponds to (Δx,Δy)=(0,2.0) cm, P10 to (2.5,0) cm, and P22 to (5.0,4.0) cm relative to P00.

To investigate the influence of cable routing on the predicted muscular response, nine distal cable-anchor positions were defined in the sagittal plane relative to the reference position P00, corresponding to the heel-marker location in the anatomical marker set. A local coordinate system was used in which Δx denotes posterior displacement and Δy denotes superior displacement. The positions were generated using increments of 2.5cm in the posterior direction and 2.0cm in the superior direction. In the Pij notation, the first index *i* indicates the number of 2.5 cm posterior increments and the second index *j* indicates the number of 2.0 cm superior increments relative to P00. The exact offsets are reported in Table 2. The spatial arrangement of these nine distal anchor positions is illustrated in Figure 3.

All configurations were evaluated under identical prescribed kinematics and the same target assistance profile. Cable trajectories were also checked to ensure that no modeled component interacted with the floor during simulation. The purpose of this analysis was not to identify a universally optimal anchor position, but to quantify the sensitivity of the predicted muscular and metabolic responses to cable-routing geometry under controlled conditions.

For consistency, the hyphenated notation used in the previous version (e.g., P–01) has been removed. All anchor configurations are now denoted uniformly as Pij (P00–P22) throughout the text, tables, and figures.

## 3. Results

The CMC simulations were used to estimate the sensitivity of predicted plantar-flexor muscle activations to variations in distal cable-anchor position during the gait cycle (0–100%). Eight major plantar-flexor muscles were analyzed: Flexor Digitorum Longus (FDL), Flexor Hallucis Longus (FHL), Lateral Gastrocnemius (LG), Medial Gastrocnemius (MG), Soleus (SOL), Peroneus Longus (PL), Peroneus Brevis (PB), and Tibialis Posterior (TP). Each simulated condition (P00–P22) represented a unique distal cable-anchor location, whereas the non-actuated condition (noAct) corresponded to the baseline gait without external cable assistance.

The following results correspond to model-derived predictions obtained under identical prescribed kinematics and identical assistance magnitude for all cable configurations. Consequently, they should be interpreted as relative differences between simulated conditions rather than as experimentally observed neuromuscular responses.

### 3.1. Muscle Activation

**Figure 4:**
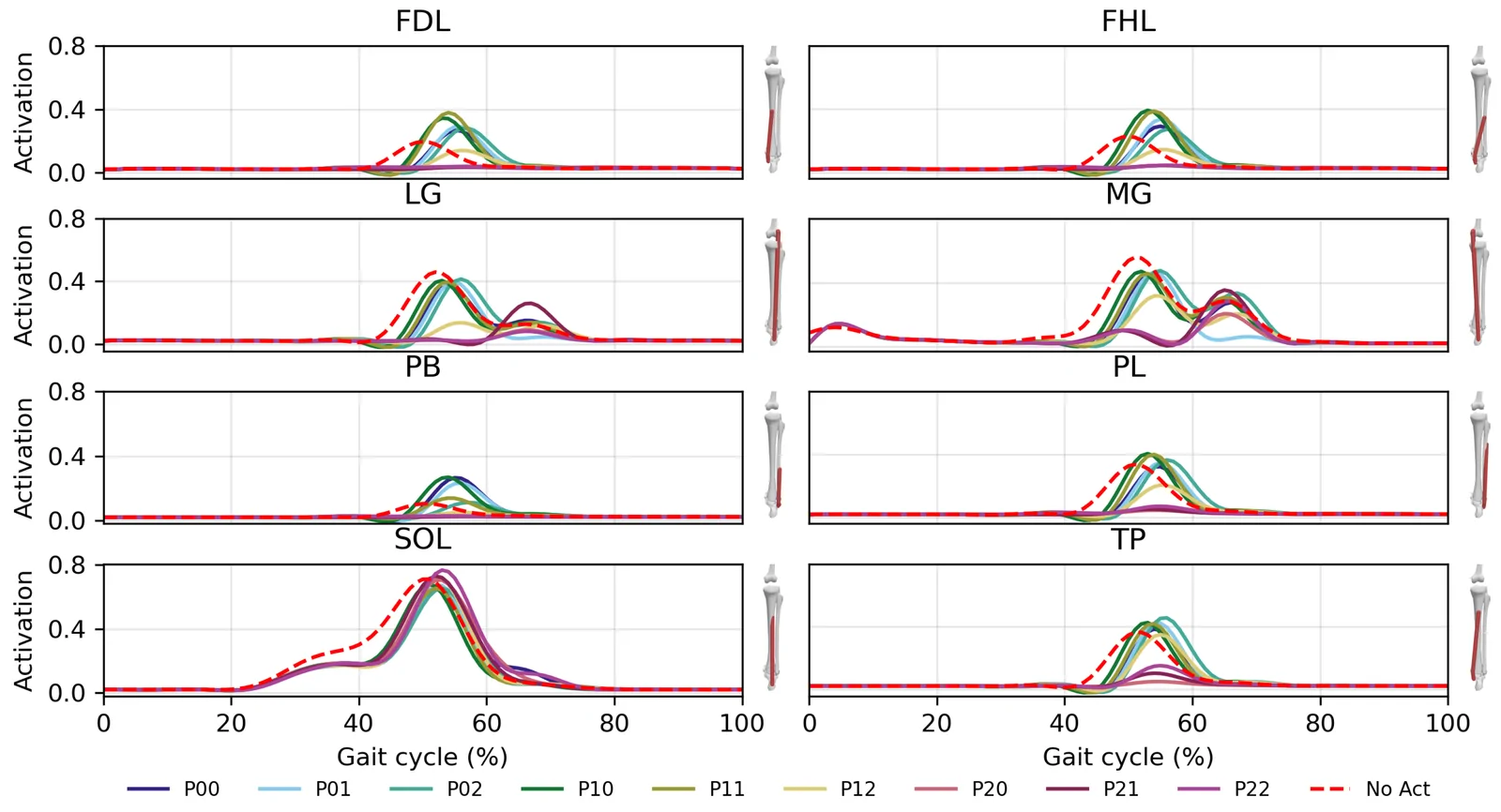
**CMC-derived predicted activation profiles for eight superficial and deep plantar-flexor muscles throughout the gait cycle.** The curves illustrate the muscle activation estimates generated by the simulation framework to reproduce the prescribed kinematics under nine different cable-anchor configurations (P00 to P22), compared with the non-actuated baseline condition (noAct). Peak activations generally occurred during late stance (40–65% of the gait cycle), corresponding to the phase in which the model predicted the greatest plantar-flexor contribution to push-off and foot stabilization. All simulations were performed using the same experimental gait dataset and identical prescribed kinematics, allowing direct comparison of the predicted effects associated with cable geometry.

Across all muscles, the predicted activation profiles exhibited temporal patterns consistent with the expected contribution of plantar-flexor muscles during gait, with relatively low activity during early stance and increased activation during mid-to-late stance associated with push-off (Figure 4).

Variations in cable-anchor position modified both the magnitude and timing of the predicted muscle activations, particularly for the gastrocnemius and soleus muscles, which are the principal contributors to ankle plantar flexion.

For both heads of the gastrocnemius, several cable configurations were associated with lower predicted peak activations compared with the non-actuated condition. The largest reductions were generally observed for P22, P01, and P00, whereas alternative configurations produced only minor changes in activation amplitude.

The soleus exhibited lower predicted activations than the noAct condition for all the simulated configurations. However, the configurations with the largest posterior offset (P20–P22) tended to exhibit higher soleus activation peaks than the configurations with smaller posterior offsets, whereas the configurations nearer the P00 heel-reference position produced the lowest predicted soleus activations during terminal stance.

The secondary plantar-flexor muscles exhibited greater sensitivity to cable placement than the gastrocnemius and soleus muscles. The Peroneus Brevis (PB) and Peroneus Longus (PL) showed pronounced activation peaks during late stance (approximately 55–65% of the gait cycle). Among these muscles, PB exhibited the greatest variability between conditions. The zero-superior-offset configurations (P00, P10, and P20) were associated with the highest predicted activation peaks (approximately 0.3–0.4), whereas the configurations with larger superior offsets generally reduced the estimated activation levels.

Among the deep posterior muscles, FDL and FHL exhibited the largest variation in predicted activation amplitudes across configurations, with peak values ranging approximately between 0.1 and 0.4. These differences suggest a redistribution of the predicted muscular contribution among muscles involved in toe flexion and foot stabilization as a consequence of changes in cable routing geometry. Activation amplitudes were generally similar to or higher than those observed in the non-actuated condition for several configurations, whereas P00, P01, and P02 tended to produce lower activation estimates.

The Tibialis Posterior (TP) showed consistently low activation levels (peak values below 0.2) across all conditions. Nevertheless, several assisted configurations exhibited slightly higher activation levels than the non-actuated condition, which is consistent with the known biomechanical role of this muscle in inversion control and medial arch stabilization.

Overall, the simulations predicted reductions in the activation of the principal plantar-flexor muscles (gastrocnemius and soleus) for all cable-anchor configurations relative to the non-actuated condition. In contrast, some secondary plantar-flexor muscles exhibited increased activation under specific cable positions, particularly in configurations located closer to the heel region. Conversely, configurations located further away from the heel generally resulted in lower activation estimates for those secondary muscles.

These findings suggest that cable-anchor location influences the distribution of the predicted muscular demand among primary and secondary plantar-flexor muscles. However, because all the results correspond to model-derived predictions obtained under prescribed kinematics, they should not be interpreted as direct evidence of physiological adaptation or clinical effectiveness during real exosuit-assisted walking.

### 3.2. Muscle Force

The following results correspond to model-derived estimates of muscle force obtained under identical prescribed kinematics and identical assistance magnitude for all cable configurations. Consequently, the reported differences should be interpreted as relative variations between simulated conditions rather than as experimentally measured muscle forces.

**Figure 5:**
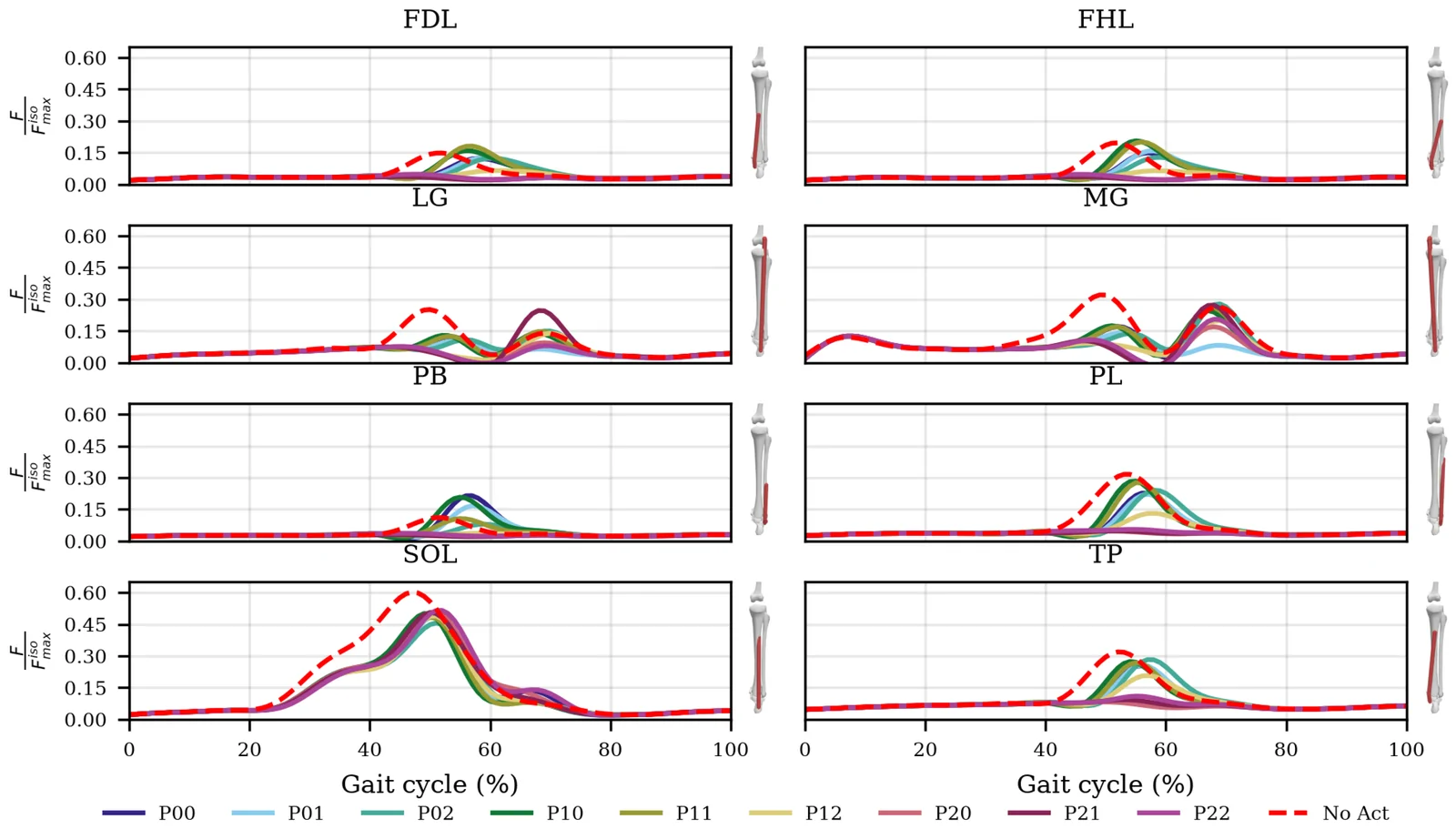
Predicted muscle force profiles of the eight plantar-flexor muscles for each cable-anchor configuration. All subfigures use the same vertical-axis range, with an upper limit of 0.65, to facilitate direct visual comparison across muscles and configurations.

Across all muscles, the non-actuated condition exhibited the characteristic temporal force profile expected during gait, with a marked increase in force generation during late stance (40–60% of the gait cycle), corresponding to the push-off phase. The introduction of simulated cable assistance modified both the timing and magnitude of the predicted force peaks, although the extent and pattern of these changes varied among muscles (Figure 5).

For the principal plantar-flexor muscles, the simulations predicted reductions in force demand during the assistance phase. In both heads of the gastrocnemius, the largest reductions in peak force were generally associated with cable configurations located further from the heel region (P02–P22). For more proximal or medial cable positions, the reductions in predicted gastrocnemius force were less pronounced.

The soleus, another major contributor to ankle plantar flexion, exhibited lower predicted force levels than the non-actuated condition across all the cable configurations. The magnitude of this reduction remained relatively similar among the configurations, although a slight increase in soleus force was observed during the end of the assistance phase for some of the most distal cable positions.

The FDL exhibited relatively low normalized force values, similar to those observed for the FHL, together with a slight delay in the force peak during stance compared with the non-actuated gait condition. Consistent with the activation results, cable configurations located in medial or heel-proximal positions tended to produce force peaks that were similar to or slightly higher than those obtained during unassisted gait, whereas configurations located further away generally produced force profiles that more closely approached the baseline condition.

The variability observed among FDL and FHL conditions was lower than that observed for the gastrocnemius or soleus muscles, suggesting a lower sensitivity of these muscles to the cable-routing modifications explored in the present simulation framework.

For the PB muscle, cable configurations located closest to the heel were associated with increases in the predicted force demand. In contrast, several medial configurations (P11 and P12) and the most distant configurations (P20, P21 and P22) exhibited noticeable reductions in force relative to the non-actuated condition.

For both PL and TP, the simulations generally predicted reductions in force production across most cable configurations, with the largest reductions again occurring for cable positions furthest from the heel region.

Overall, the simulations suggest that cable-anchor geometry may redistribute the predicted force demand among plantar-flexor muscles during push-off, increasing the contribution of some muscles while reducing the demand on others depending on the selected cable routing strategy.

Because muscle force estimates were not validated experimentally, these findings should be interpreted as relative model predictions useful for comparing cable configurations under controlled simulation conditions rather than as direct measurements of physiological muscle loading during exosuit-assisted walking.

#### Influence of the Anchor Point Position

As discussed in the preceding sections, the simulated cable assistance was generally associated with reductions in the predicted muscle activations and muscle force demands of the principal plantar-flexor muscles. To obtain an aggregate measure of these effects across the gait cycle and to quantitatively compare the different cable configurations, the mechanical impulse was computed according to Equation (9). Specifically, the mechanical impulse generated by each plantar-flexor muscle was evaluated for every cable-anchor configuration and compared with the reference non-actuated gait condition:

(9) $$\Delta {\bar{P}}_{m}=\int_{t_{i}}^{t_{f}}{\vec{F}}_{m}\;dt$$

where ${\bar{P}}_{m}$ denotes the mechanical impulse generated by muscle *m*; ${\vec{F}}_{m}$ corresponds to the predicted muscle force; and $t_{i}$ and $t_{f}$ represent the initial and final instants of the gait cycle, respectively.

The variation in mechanical impulse between assisted and non-assisted conditions provides an integrated measure of the predicted redistribution of muscular demand associated with the different cable geometries explored in the simulations.

**Figure 6:**
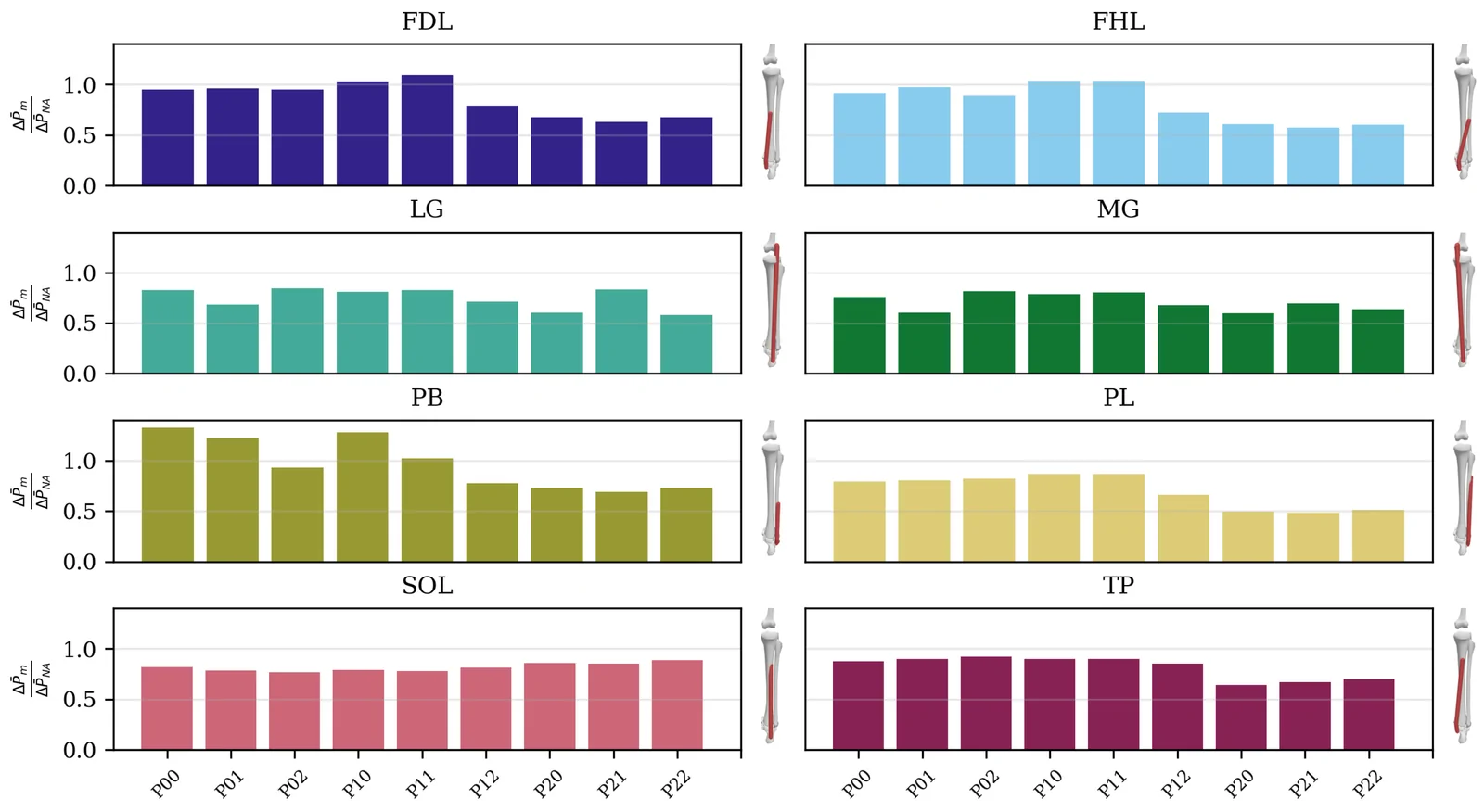
Relative mechanical impulse of each plantar-flexor muscle (${\bar{P}}_{m}$) for the different cable-anchor configurations with respect to the non-actuated gait condition (${\bar{P}}_{NA}$). Mechanical impulse was calculated through numerical integration of the predicted muscle force over the gait cycle.

Figure 6 presents the normalized mechanical impulse associated with each cable-anchor configuration. Values greater than unity indicate that the predicted mechanical impulse of a given muscle exceeded that observed in the non-actuated condition, whereas values below unity indicate a reduction in the cumulative force contribution over the gait cycle.

The principal plantar-flexor muscles (LG, MG and SOL) generally exhibited reduced mechanical impulse values relative to the baseline gait condition across most cable configurations. These reductions are consistent with the partial contribution of the simulated assistance torque to the ankle plantar-flexion moment and with the lower force demands predicted for these muscles during push-off.

A similar tendency was observed for several of the secondary plantar-flexor muscles in many of the simulated configurations. Nevertheless, certain cable locations produced normalized impulse values above unity for specific muscles, indicating that some cable geometries may redistribute the muscular demand towards secondary contributors even when the overall plantar-flexion demand decreases (see Figure 5).

Among the evaluated configurations, P20 was associated with one of the most consistent reductions in mechanical impulse across the majority of the plantar-flexor muscles analyzed. However, this tendency was not observed uniformly across all muscles or all biomechanical variables considered in this study.

Importantly, no single cable-anchor configuration simultaneously minimized all predicted biomechanical metrics. While some configurations produced lower muscle activations, others reduced cumulative impulse or metabolic demand more effectively. Consequently, the preferred cable position appears to depend on the selected optimization objective and on the specific biomechanical outcome of interest rather than on the existence of a universally optimal anchor location.

Because these results correspond to model-derived predictions obtained under prescribed kinematics, they should not be interpreted as evidence of physiological adaptation or as direct recommendations for clinical implementation. Instead, they provide a preliminary assessment of the sensitivity of the predicted muscular responses to changes in cable-routing geometry within the proposed simulation framework.

### 3.3. Metabolic Cost

**Figure 7:**
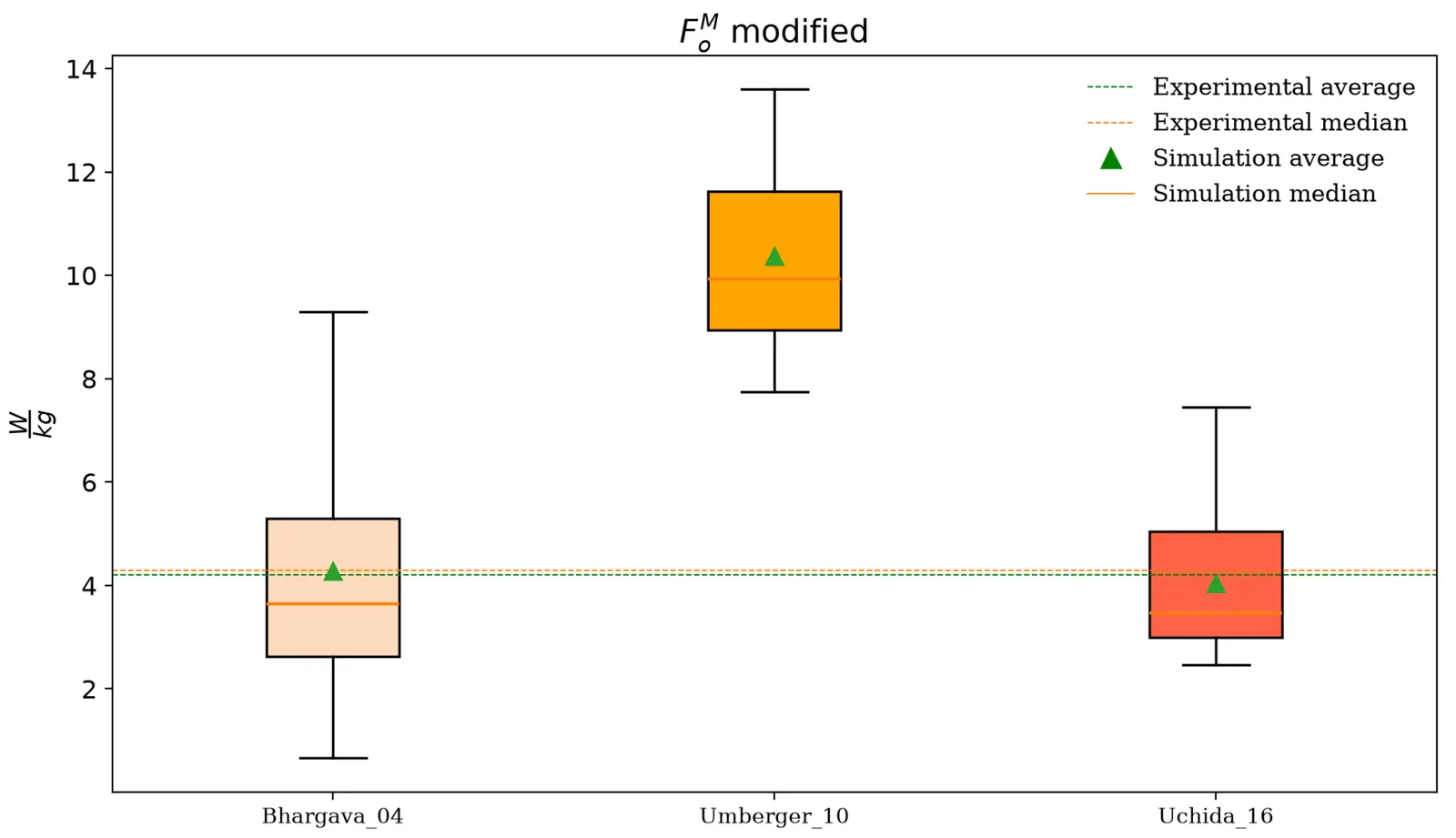
Comparison between experimentally measured and simulated baseline metabolic cost for the Bhargava, Umberger, and Uchida models. The figure reports both the experimental average and median, together with the simulated average and median, to provide a more complete representation of the central tendency of the data.

To perform a baseline comparison of the metabolic models described in the previous section, the simulated metabolic estimates were compared with the experimentally measured metabolic cost obtained from indirect calorimetry using Equation (4) (Figure 7). The purpose of this comparison was not to validate the metabolic models, but rather to assess whether the predicted values were within a physiologically plausible range for the baseline walking condition.

Regarding the model proposed by [39], and as illustrated in Figure 7, this formulation tended to overestimate metabolic power relative to the experimental baseline measurements. This observation is consistent with previous reports in the literature. For example, ref. [20] compared several metabolic formulations in healthy subjects and reported that the models proposed in [39,40] generally produce higher metabolic estimates than those observed experimentally.

The different absolute estimates obtained with the Bhargava, Umberger, and Uchida formulations reflect differences in their underlying phenomenological assumptions and in the representation of activation, maintenance, muscle shortening/lengthening, and mechanical-work terms. Because several components differ simultaneously among the models, the higher estimate obtained with the Umberger formulation should not be attributed to a single mechanism such as the treatment of eccentric work [20].

The relative differences were calculated as:

(10) $$\Delta_{\exp}=\left(\frac{{\bar{E}}_{\mathrm{sim},P_{XY}}-{\bar{E}}_{\exp}}{{\bar{E}}_{\exp}}\right)\times 100,$$

where ${\bar{E}}_{\mathrm{sim},P_{XY}}$ is the mean metabolic cost obtained from the simulation for the cable-anchor position $P_{XY}$, and ${\bar{E}}_{\exp}$ is the mean metabolic cost obtained from the experimental data. The experimental metabolic cost was calculated using only the middle portion of the gait trial, excluding the first and last 2 min of the recording:

(11) $$\Delta_{\mathrm{noAct}}=\left(\frac{{\bar{E}}_{\mathrm{sim},P_{XY}}-{\bar{E}}_{\mathrm{noAct}}}{{\bar{E}}_{\mathrm{noAct}}}\right)\times 100.$$

where ${\bar{E}}_{\mathrm{sim},P_{XY}}$ and ${\bar{E}}_{\mathrm{noAct}}$ are the mean simulated metabolic cost values obtained according to Equation (3).

Table 3 summarizes the estimated metabolic power across the experimental baseline, the simulated non-actuated condition, and the active cable-anchor configurations ($P_{XY}$). Using the absolute metabolic-power values reported in the table, the simulated non-actuated condition was 4.283W/kg, corresponding to a 1.855% difference from the experimental baseline (4.205W/kg). Across the active cable-anchor configurations, the predicted metabolic power relative to the experimental baseline ranged from a 0.856% reduction (P20) to a 6.278% increase (P00). Relative to the simulated non-actuated condition, the predicted changes ranged from a 2.659% reduction (P20) to a 4.333% increase (P00).

The predicted energetic response varied across the different cable-anchor locations. P01, P10, P12, and P20 produced modest reductions relative to the experimental baseline, whereas P00, P02, P11, P21, and P22 produced small-to-moderate increases. Relative to the simulated non-actuated condition, P01, P10, P11, P12, and P20 showed reductions of approximately 1.5–2.7%, whereas P00, P02, P21, and P22 showed increases of approximately 3.9–4.3%. These results indicate that the predicted energetic response is sensitive to anchor location within the present model. In particular, changes in anchor position modify the cable orientation and instantaneous moment arm and therefore the tensile force required to reproduce the same prescribed ankle-assistance torque.

Although the largest differences across configurations remained modest, they should be interpreted cautiously because they may fall within the numerical variability and inherent uncertainty associated with musculoskeletal and metabolic-cost modeling. Direct experimental validation of the predicted metabolic changes under active assistance was not possible because experimental metabolic measurements were not available for the individual cable-anchor configurations. Therefore, the predicted metabolic power values should be interpreted primarily as a model-based sensitivity measure of the effect of cable-anchor geometry rather than as direct estimates of experimentally achievable metabolic reductions.

Overall, P20 produced the lowest predicted metabolic power among the evaluated assisted configurations and the largest reduction relative to both the experimental baseline and the simulated non-actuated condition. In contrast, P00 produced the highest predicted metabolic power and the largest increase relative to both reference conditions. These findings support the use of the model as a preliminary screening tool for configuration-dependent energetic sensitivity; however, they do not establish experimentally validated metabolic benefits or a clinically optimal anchor location.

## 4. Discussion

In general, the simulations predicted lower activation levels for the principal plantar-flexor muscles under most assisted configurations compared with the non-actuated gait condition. However, configurations with smaller offsets from the P00 heel-reference position were associated with greater activation of several plantar-flexor muscles and with delayed activation peaks during the gait cycle. In contrast, configurations with larger offsets from P00 generally produced lower activation estimates than those obtained in the non-assisted condition.

In addition to changes in muscle activation, variations in cable position were also associated with modifications in the predicted muscle force and metabolic responses. In particular, some configurations with larger offsets from the P00 heel-reference position tended to reduce the predicted plantar-flexor forces and were associated with lower metabolic estimates, whereas several smaller-offset configurations exhibited smaller energetic changes. Taken together, these findings suggest that cable-anchor location is associated with substantial changes in the predicted muscular and energetic responses generated by the simulation framework. Importantly, the magnitude and direction of these effects depended on the biomechanical metric considered and on the specific cable geometry evaluated.

The finding that predicted muscle activation during assisted walking was generally lower than during non-assisted walking is consistent with previous studies reporting reductions in plantar-flexor muscle activity with ankle exoskeleton assistance in cerebral palsy [41,42,43]. In contrast to studies describing relatively uniform reductions in plantar-flexor activity, the present results suggest heterogeneous responses across cable configurations, highlighting the sensitivity of the predicted muscular response to cable placement [4,43]. These differences may be associated with variations in exosuit design, mechanical advantage, and assistance delivery associated with specific cable geometries.

Moreover, by explicitly quantifying muscle force estimates together with metabolic power, the proposed framework provides additional biomechanical information that is largely absent from previous studies, which have primarily focused on spatiotemporal gait parameters or muscle activation alone [44,45]. Rather than identifying a clinically optimal cable configuration, the present study contributes to understanding how cable geometry may influence the predicted biomechanical response under controlled simulation conditions.

Consistent with this interpretation, systems capable of modulating ankle assistance across multiple degrees of freedom have reported heterogeneous muscle activation responses, particularly when assistance is delivered through different cable configurations [46]. Together with the present findings, these studies support the need for systematic evaluation of cable placement when designing and tuning ankle exosuits for gait assistance.

The observed sensitivity of predicted muscle activation and force patterns to cable position has important implications for the preliminary design of ankle exosuits. While most cable configurations reduced the predicted activation and force demands of the principal plantar-flexor muscles, certain configurations with larger offsets from P00 were associated with larger reductions in gastrocnemius force and activation, whereas configurations nearer the heel-reference position tended to reduce soleus demand during terminal stance more effectively.

However, these reductions were frequently accompanied by increased predicted activation and force demand in secondary plantar-flexor and stabilizing muscles, particularly the peroneal muscles, for configurations with smaller offsets from P00. This redistribution of the predicted muscular demand suggests the existence of trade-offs between unloading the principal push-off muscles and increasing the contribution of muscles involved in frontal-plane stability and foot control.

Consequently, the selection of cable geometry should not rely exclusively on maximizing plantar-flexor unloading but should instead consider the broader distribution of muscular demand generated by the assistance strategy. These findings support the potential value of adjustable cable geometries capable of accommodating different biomechanical objectives and patient-specific requirements.

The reductions in mechanical impulse predicted for the principal plantar-flexor muscles are consistent with the partial contribution of the simulated assistance torque to the ankle plantar-flexion moment. However, increases in mechanical impulse were observed for some muscles and cable configurations, suggesting that cable routing may redistribute rather than simply reduce muscular demand.

Importantly, no single cable-anchor configuration simultaneously minimized all biomechanical metrics considered in this study. Some configurations reduced predicted muscle activation more effectively, whereas others were associated with lower cumulative impulse or lower predicted metabolic cost. Consequently, the preferred cable geometry appears to depend on the selected optimization objective rather than on the existence of a universally optimal anchor location.

The transferability of the proposed workflow to heterogeneous CP populations depends on the interaction between participant-specific gait kinematics, anthropometry, muscle–tendon properties, and the underlying model topology. For participants whose gait deviations can be represented within the existing degrees of freedom and joint definitions of Gait2392, the workflow could in principle be applied after recalibration using participant-specific anthropometry and kinematics. Additional adaptation of muscle–tendon parameters, such as maximum isometric force, optimal fiber length, or tendon slack length, may also be required when clinically relevant differences in muscle strength, contracture, or muscle–tendon properties are present. Representations of spasticity or altered reflex behavior constitute possible future extensions rather than features implemented or validated in the present framework.

In contrast, participants with substantial structural deformities or skeletal malalignment, such as pronounced tibial torsion or fixed multiplanar foot deformities, may require more fundamental model modifications, including participant-specific joint axes, muscle paths, or wrapping surfaces. In such cases, simple parameter recalibration of the generic model may not be sufficient.

It is therefore important to distinguish between the transferability of the experimental–computational workflow and the generalizability of the present quantitative predictions: the workflow may be applicable to other participants after appropriate recalibration, whereas the present results remain specific to this participant and to the modeling assumptions used. Overall, the framework is intended to support preliminary design comparisons rather than to identify clinically optimal exosuit configurations.

### Limitations and Future Work

The present study should be interpreted as a single-participant proof-of-concept investigation. Consequently, the reported findings cannot be generalized to the broader cerebral palsy population and should not be interpreted as universal recommendations for ankle exosuit design or clinical implementation. Future studies should apply the proposed framework to larger and more heterogeneous cohorts in order to evaluate inter-subject variability and determine whether consistent biomechanical trends emerge across different clinical presentations.

The gait data used to construct the musculoskeletal model were acquired while the participant was wearing a clinically prescribed passive ankle–foot orthosis (AFO), which forms part of the subject’s regular clinical management. Because the mechanical contribution of the AFO was not characterized or modeled separately, the inverse-dynamics ankle moment represents the net moment associated with AFO-supported gait and cannot be decomposed into independent biological and orthotic contributions. Consequently, the simulated conditions should not be interpreted as unbraced pathological gait. Future studies should include measurements with and without orthotic support, or explicit mechanical characterization of the AFO, to quantify these contributions separately.

Experimental validation was restricted to an aggregate metabolic-power measurement for the baseline non-assisted condition. The simulated non-actuated metabolic power differed from the experimental baseline by 0.0031W/kg (0.08%), which is reported as a physiological plausibility check rather than as comprehensive model validation. Because no time-resolved experimental metabolic signal, electromyographic recordings, or in vivo individual muscle-force measurements were available, RMSE, correlation coefficients, or equivalent physiological agreement metrics could not be calculated for these outputs. No physiological measurements were available for the simulated exosuit-assisted configurations; consequently, muscle activation, muscle force, and assisted-condition metabolic-power estimates should be interpreted as model-derived predictions rather than experimentally validated quantities. Future work should incorporate electromyographic recordings, repeated metabolic measurements, and assisted walking trials with physical exosuit prototypes to assess the physiological validity and repeatability of the predicted responses.

The musculoskeletal simulations relied on an anthropometrically scaled version of the OpenSim Gait2392 model originally developed for healthy adults. Although the standard marker-based scaling procedure adapted segment dimensions to the participant and its marker-fit error is now reported, no functional calibration of joint centers or imaging-based verification of individual skeletal topology was performed. Therefore, the reported RMS marker error cannot be interpreted as an estimate of the error in joint-center locations or muscle moment arms. In addition, no representation of spasticity, muscle contractures, altered tendon properties, or other subject-specific neuromuscular impairments associated with cerebral palsy was included. Future studies should incorporate functional joint calibration and, where clinically warranted, medical-imaging-based or otherwise subject-specific musculoskeletal geometry to quantify the sensitivity of the predicted responses to these anatomical assumptions.

The present investigation considered a fixed assistance magnitude and cable displacements restricted to the sagittal plane. Consequently, the influence of assistance level, mediolateral cable placement, and frontal-plane stability effects were not investigated. Future studies should incorporate three-dimensional cable-routing strategies, variable assistance magnitudes, and dedicated stability metrics to enable more comprehensive optimization analyses.

Finally, the results were obtained using a deterministic simulation framework applied to a single experimental dataset. No statistical analysis, uncertainty quantification, sensitivity analysis, or repeatability assessment was performed. The present study was not intended to establish a robust ranking of cable-anchor configurations; muscle-strength scaling, tendon properties, assistance magnitude, and gait-cycle selection were kept fixed so that cable geometry remained the primary variable under investigation. Therefore, the observed differences should be interpreted as exploratory model predictions under controlled conditions rather than statistically validated effects. Future work should systematically vary relevant assumptions, including muscle-strength scaling, tendon compliance, assistance magnitude, and gait-cycle selection, to determine whether the relative differences among cable-anchor configurations remain stable.

## 5. Conclusions

The present work investigated the influence of distal cable-anchor location on the predicted muscular and energetic responses associated with ankle exosuit assistance in a child with spastic hemiplegic cerebral palsy using an experimental–computational simulation framework.

Overall, most cable configurations were associated with reductions in the predicted activation and force demand of the principal ankle plantar-flexor muscles, particularly the soleus and both heads of the gastrocnemius. However, the magnitude and distribution of these effects depended strongly on the selected cable geometry and on the biomechanical metric considered.

Predicted metabolic power varied across assisted configurations, ranging from a 2.66% reduction to a 4.33% increase relative to the simulated non-actuated condition. Relative to the experimental baseline, the corresponding range was a 0.86% reduction to a 6.28% increase. Because metabolic measurements were obtained experimentally only for the baseline condition, these assisted-condition differences remain model-derived and were interpreted primarily as indicators of sensitivity to cable geometry rather than as evidence of experimentally demonstrated functional or clinical benefit.

Importantly, no single cable-anchor configuration simultaneously minimized all biomechanical outcomes investigated in this study. While some configurations reduced predicted muscle activation more effectively, others were associated with lower cumulative impulse or lower predicted metabolic demand. These findings suggest that cable placement should be regarded as a multi-objective design problem rather than as the identification of a universally optimal attachment location.

Finally, although the present study should be interpreted as a proof-of-concept investigation based on a single participant and model-derived predictions, it demonstrates the sensitivity of the proposed framework to cable geometry and provides a transferable methodology that may support future participant-specific exosuit design and optimization studies.

## Figures and Tables

**Table 1:** Peak and root-mean-square (RMS) cable forces calculated over the complete gait cycle for each distal anchor configuration.

Configuration	Peak Force (N)	RMS Force (N)
P00	250.06	79.41
P01	250.54	79.31
P02	251.09	81.21
P10	182.54	57.81
P11	179.96	57.63
P12	177.16	55.00
P20	144.51	46.26
P21	141.27	44.32
P22	137.81	43.43

**Table 2:** Distal cable-anchor offsets relative to the reference heel-marker position P00. Positive Δx indicates posterior displacement and positive Δy indicates superior displacement.

Configuration	Δx (cm)	Δy (cm)
P00	0.0	0.0
P01	0.0	2.0
P02	0.0	4.0
P10	2.5	0.0
P11	2.5	2.0
P12	2.5	4.0
P20	5.0	0.0
P21	5.0	2.0
P22	5.0	4.0

**Table 3:** Experimental and simulated metabolic power for the experimental baseline, the simulated non-actuated condition, and the cable-anchor configurations: $\Delta_{\exp}$ denotes the relative difference from the experimental baseline and $\Delta_{\mathrm{noAct}}$ the relative difference from the simulated non-actuated condition. Values for P00–P22 are model-derived predictions and were not experimentally validated under assisted conditions.

Condition	Metabolic Power (W/kg)	$\Delta_{\exp}$ (%)	$\Delta_{\mathrm{noAct}}$ (%)
Experimental baseline	4.205	–	–
Simulated non-actuated	4.283	+1.855	–
P00	4.469	+6.278	+4.333
P01	4.178	−0.642	−2.451
P02	4.449	+5.803	+3.880
P10	4.189	−0.380	−2.182
P11	4.220	+0.357	−1.461
P12	4.185	−0.476	−2.295
P20	4.169	−0.856	−2.659
P21	4.461	+6.088	+4.160
P22	4.462	+6.112	+4.202

## Data Availability

The raw data supporting the conclusions of this article will be made available by the authors upon request.

## Abbreviations

The following abbreviations are used in this manuscript:

AFO	ankle–foot orthosis
CMC	Computed Muscle Control
CP	cerebral palsy
FDL	Flexor Digitorum Longus
FHL	Flexor Hallucis Longus
LG	Lateral Gastrocnemius
MG	Medial Gastrocnemius
MC	Metabolic Cost
PB	Peroneus Brevis
PL	Peroneus Longus
RRA	Residual Reduction Algorithm
SOL	Soleus
TP	Tibialis Posterior
$VO_{2}$	oxygen uptake
$VCO_{2}$	carbon dioxide production
